# FGF signaling in cranial suture development and related diseases

**DOI:** 10.3389/fcell.2023.1112890

**Published:** 2023-06-01

**Authors:** Xiaolei Zhao, Shannon Erhardt, Kihan Sung, Jun Wang

**Affiliations:** ^1^ Department of Pediatrics, McGovern Medical School, The University of Texas Health Science Center at Houston, Houston, TX, United States; ^2^ MD Anderson Cancer Center and UT Health Graduate School of Biomedical Sciences, The University of Texas, Houston, TX, United States; ^3^ Department of BioSciences, Rice University, Houston, TX, United States

**Keywords:** suture mesenchymal stem cell, neural crest, cranial suture, repair, craniosynostosis, FGF signaling

## Abstract

Suture mesenchymal stem cells (SMSCs) are a heterogeneous stem cell population with the ability to self-renew and differentiate into multiple cell lineages. The cranial suture provides a niche for SMSCs to maintain suture patency, allowing for cranial bone repair and regeneration. In addition, the cranial suture functions as an intramembranous bone growth site during craniofacial bone development. Defects in suture development have been implicated in various congenital diseases, such as sutural agenesis and craniosynostosis. However, it remains largely unknown how intricate signaling pathways orchestrate suture and SMSC function in craniofacial bone development, homeostasis, repair and diseases. Studies in patients with syndromic craniosynostosis identified fibroblast growth factor (FGF) signaling as an important signaling pathway that regulates cranial vault development. A series of *in vitro* and *in vivo* studies have since revealed the critical roles of FGF signaling in SMSCs, cranial suture and cranial skeleton development, and the pathogenesis of related diseases. Here, we summarize the characteristics of cranial sutures and SMSCs, and the important functions of the FGF signaling pathway in SMSC and cranial suture development as well as diseases caused by suture dysfunction. We also discuss emerging current and future studies of signaling regulation in SMSCs.

## 1 Introduction

**FIGURE 1:**
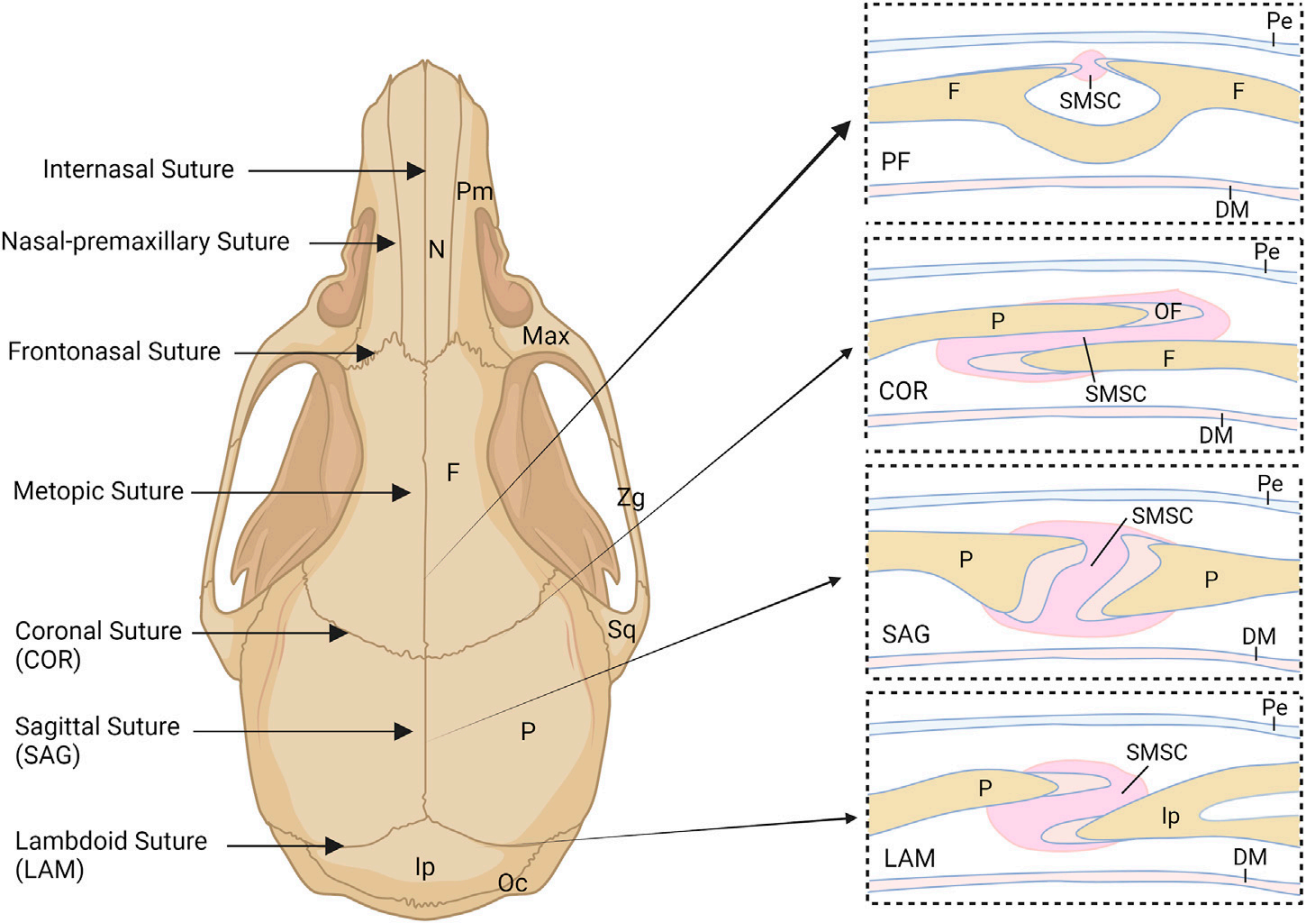
Schematic illustration of the murine skull and cranial sutures. COR, coronal suture; DM, dural mater; F, frontal bone; Ip, interparietal bone; LAM, lambdoid suture; Max, maxilla; N, nasal bone; OF, osteogenic front; Oc, occipital bone; P, parietal bone; Pe, periosteum; PF, posterior frontal suture; Pm, premaxilla; SAG, sagittal suture; SMSC, suture mesenchymal stem cell; Sq, squamosal; Zg, zygomatic.

Different from long bones, which are formed through endochondral ossification, most cranial bones are formed through intramembranous ossification directly from mesenchymal cells without a cartilaginous template (Ishii et al., 2015). As shown in Figure 1, these cranial bones are connected by fibrous joints, known as cranial sutures, consisting of fibrous tissues with mesenchyme, two osteogenic fronts (OFs) of the approximating bone plates, underlying dura mater, and overlying periosteum. Notably, the OFs of the bone plates of the coronal and lambdoid sutures partially overlap, whereas the OFs of the metopic and sagittal sutures abut from end to end (Lenton et al., 2005). Cranial sutures provide postnatal locomotive shock absorption and allow joint mobility during feeding (White et al., 2021). They also function as an intramembranous bone growth site for cranial bone expansion during embryogenesis and postnatal craniofacial growth (Opperman, 2000). Furthermore, the cranial suture provides a niche for mesenchymal stem cells (Zhao et al., 2015), called suture mesenchymal stem cells (SMSCs), which maintain suture patency during craniofacial development and craniofacial bones homeostasis, repair and regeneration. In mice, most sutures remain patent throughout the lifetime except for the posterior frontal suture (PFS) located between the frontal bones (Sahar et al., 2005). In humans, the metopic suture (also known as the frontal suture) fuses between 3 and 8 months of age, whereas other cranial sutures fuse between 20 and 30 years, and facial sutures fuse after 50 years (Vu et al., 2001; Weinzweig et al., 2003; White et al., 2021). Cranial suture patency is important for allowing the skull to grow in concert with the development of the brain during childhood. Aberrant development of cranial sutures leads to various congenital diseases such as sutural agenesis and craniosynostosis (Cohen, 1993; Barnes, 2012; Ishii et al., 2015). Despite the considerable significance of cranial sutures and SMSCs, they have remained poorly understood. Recently, however, multiple single cell RNA-sequencing (scRNA-seq) studies of frontal and coronal suture tissues have characterized SMSC populations to a certain extent (Holmes et al., 2020; Farmer et al., 2021; Holmes et al., 2021).

The development of cranial sutures and SMSCs involves multiple factors including Twist (Carver et al., 2002; Yoshida et al., 2005; Ting et al., 2009), fibroblast growth factor (FGF) ligands and receptors (FGFRs) (Johnson et al., 2000; Rice et al., 2000; Greenwald et al., 2001; Marie et al., 2005; Wang et al., 2005; Robin et al., 2011), Msx1/2 (Liu et al., 1999; Merrill, 2005), TCF12 (Ting et al., 2022), Axin2 (Yu et al., 2005; Behr et al., 2013) and Gli1 (Zhao et al., 2015; Yu et al., 2021; Jing et al., 2022), as well as signaling such as Hedgehog (Kim et al., 1998; Jenkins et al., 2007; Rice et al., 2010), wingless-related integration site (Wnt) (Behr et al., 2010), Notch (Liu et al., 2017), transforming growth factor/bone morphogenetic protein (TGF/BMP) (Opperman et al., 1997; Clendenning and Mortlock, 2012), Hippo-Yap (Dong et al., 2021; Zhao et al., 2022), and mechanical signaling (Herring, 2008; Wang et al., 2014). Among them, the fundamental FGF signaling has been shown to play a pivotal role in maintaining cranial suture patency and SMSC development. In humans, FGFR mutations have been associated with craniosynostosis in patients with Apert and Crouzon syndromes (*FGFR2* gain-of-function mutation) and Muenke syndrome (*FGFR3* gain-of-function mutation) (Wilkie et al., 1995; Doherty et al., 2007). In addition, ectopic *FGF2* expression in mouse embryos was shown to lead to coronal suture synostosis (Mathijssen et al., 2000). In humans, the FGF family includes 22 ligands, 4 of which are not secreted and act intracellularly (Olsen et al., 2003). The remaining 18 ligands (FGF1-10 and FGF16-23) act through 4 transmembrane tyrosine kinase receptors (FGFR1-4) and are involved in multiple cell functions, such as cellular stemness, proliferation, differentiation and regeneration (Sasaki et al., 2006; Gotoh, 2009; Li et al., 2010; Mossahebi-Mohammadi et al., 2020; Farooq et al., 2021; Kumar et al., 2021). However, our understanding of the precise role of FGF-mediated signaling in cranial suture development and related diseases is limited. In this review, we summarize the most up-to-date advances in the cranial suture and SMSC research. We provide an overview of the FGF pathway and its crosstalk with other signals in cranial suture development in different experimental models to provide deeper insight into the mechanisms of the FGF pathway in cranial suture development and related diseases. Finally, we discuss areas for future studies of the regulation of FGF signaling in cranial suture development and diseases, such as craniosynostosis.

## 2 Cranial sutures and SMSCs in cranial bone formation and repair

Most cranial bones, such as the nasal bone, frontal bone, and part of the interparietal bone, are derived from neural crest (NC) cells, whereas the parietal bone and most of the occipital bones originate from paraxial mesoderm cells (Jiang et al., 2002; Yoshida et al., 2008; Doro et al., 2019). Reports have shown that the intrinsic proliferation and osteogenic abilities of NC-derived mesenchyme are higher than those of mesoderm-derived (Jiang et al., 2002; Doro et al., 2019; Siismets and Hatch, 2020; Srinivasan et al., 2020). The cranial sutures connect the separate cranial bones as a rigid entity to support the craniofacial structures and to provide a protective cavity for the brain (Li et al., 2021). The major sutures of the skull vault include the metopic (frontal/interfrontal) suture located between the two frontal bone plates, the sagittal suture located between the two parietal bone plates, the coronal suture located between the frontal bone and parietal bone, and the lambdoid suture located between the parietal bone and occipital bone (Li et al., 2021) (Figure 1). The sutures between cranial bones are also populated by mesenchymal cell populations from different embryological origins. For example, the metopic and predominant sagittal sutures are derived from NC cells and the coronal suture is derived from paraxial mesoderm, confirmed by Jiang et al. and Lenton et al. (Jiang et al., 2002; Lenton et al., 2005). However, Doro et al. recently found that both the NC and mesoderm contribute to the coronal suture (Doro et al., 2019). The origin of the lambdoid suture remains unclear. The results of lineage tracking experiments have indicated that the underlying dura mater surrounding the cerebral hemispheres but not the midbrain or hindbrain originates from NC cells (Jiang et al., 2002). Different embryonic origins may result in distinct properties of SMSCs and their derivatives within various sutures.

Several populations of SMSCs in cranial sutures, including Gli1+ (Zhao et al., 2015; Yu et al., 2021), Axin2+ (Maruyama et al., 2016), Prrx1+ (Wilk et al., 2017), and Ctsk+ (Debnath et al., 2018; Otaify et al., 2018) mesenchymal cells, have been identified and proposed as major populations of SMSCs. The characteristics of these four populations of SMSCs have been well summarized in reviews by Doro et al. (Doro et al., 2017) and Li et al. (Li et al., 2021). In general, these four subpopulations of SMSCs have similar but not identical characteristics. They all possess self-renewal and multi-lineage differentiation abilities in mice (except for Prrx1+ SMSCs which were tested only for osteogenic differentiation) and are maintained abundantly in the cranial suture for more than 1 year in mice (excluding the Prrx1+ SMSCs population, which significantly and continuously decreased with age from 8 to 32 weeks of age), and they contribute to calvarial bone injury repair. Additionally, in lineage tracking studies in mice, the subpopulations of SMSCs showed different functions and different abilities to generate calvaria tissues *in vivo.* Gli1+ SMSCs and their derivatives were detectable in cranial suture mesenchyme, periosteum, dura mater, and quite a few osteocytes in the calvaria bones (Zhao et al., 2015). Notably, the Axin2-expressing cells and their derivatives remained detectable in the middle of the suture, and the population continued to increase in all sutures, except for the PFS (Maruyama et al., 2016). Ctsk + SMSCs and their derivatives were detectable in the cranial suture, periosteum, dura mater and bone marrow cavity of the calvarium, and also contribute to the intramembranous bone formation (Debnath et al., 2018).

In addition to the above SMSC populations, Holmes et al. recently found that Hhip, an inhibitor of Hedgehog signaling, marks a new mesenchymal population that persists only in the coronal suture, although it is also enriched in the OFs of other skull sutures (Holmes et al., 2021). Hhip distinguishes the coronal suture mesenchyme from other skull sutures. Hhip+ populations are highly enriched in the suture and can not differentiate rapidly to osteoblasts during early postnatal periods. After 90 days of tracking in mouse, Hhip-labeled cells were incorporated as osteoblasts and osteocytes in the frontal and parietal bones, but most of them remained in the coronal suture mesenchyme. Loss of Hhip population in the coronal suture resulted in apposed osteogenic fronts and depleted suture mesenchyme at E18.5 mouse embryos (Holmes et al., 2021). Farmer et al. identified a Six2+ osteoprogenitor population in the coronal suture by performing scRNA-seq of coronal suture tissues (Farmer et al., 2021). The Six2 population contributed extensively to the mesenchyme of coronal sutures and to the osteocytes of frontal and parietal bones that are close to the suture. However, it remains largely unknown whether these 6 subpopulations overlap in identity and function, how they interact among different subpopulations of SMSCs, and how these interactions contribute to craniofacial bone development, homeostasis, repair, regeneration and diseases.

Consistent with the self-renewal and multi-lineage differentiation abilities of SMSCs, they have been shown to play indispensable roles in suture patency, injury repair and tissue regeneration. Using *Gli1-Cre* ^*ERT2*^ *; R26-tdTomato* ^*flox*^ mice in which Gli1+ cells and their derivatives could be labelled with tdTomato, Zhao et al. found that Gli1 Lineage cells in the suture mesenchyme of the sagittal suture can be promptly activated to proliferate within 24 h after injury by drilling a 1 mm diameter hole 2 mm away from the sagittal suture in the parietal bone, and the majority of the cells within the injured area were Gli1 lineage after 2 weeks post-injury (Zhao et al., 2015). One month post-injury, the dura mater, periosteum and many osteocytes in the repair site were robustly labelled with tdTomato indicating that Gli1 Lineage contribute to injury repair of the calvarial bone (Zhao et al., 2015). Their further study displayed that a piece of transplanted parietal bone containing the sagittal suture can generate new dura mater and periosteum in the 4 mm^2^ defect region of the parietal bone of recipient nude mice, and can merge with the host bone after 1 month of transplantation, while the parietal bones without a portion of the suture fail to do so (Zhao et al., 2015). Deleting Gli1 Lineage using cre-inducible diphtheria toxin A (DTA) in one-month-old *Gli1-Cre* ^*ERT2*^ *;DTA* ^*flox/flox*^ mice led to coronal and frontal-premaxilla suture fusion after 1-month induction and all craniofacial sutures fusion after 2-month induction with skull growth arrest and osteoporosis. This further revealed the indispensable roles of the Gli1+ population of SMSCs in suture patency maintenance, efficient cranial bone repair and regeneration (Zhao et al., 2015).

Similar to the Gli1+ SMSCs, Axin2+ SMSCs also rapidly respond to calvarial bone injury and directly contribute to calvarial bone regeneration in response to injury in mice (Maruyama et al., 2016). Additionally, Axin2 plays an important role in maintaining suture patency (Di Pietro et al., 2020), and the targeted disruption of Axin2 in mice induces malformations of skull structures, a phenotype resembling craniosynostosis in humans (Yu et al., 2005). Similarly, Wilk et al. found that Prrx1+ SMSCs contributed to calvarial bone repair and regeneration in both NC-derived (frontal) and mesoderm-derived (parietal) bones in mice (Wilk et al., 2017). Unlike Gli1+ and Axin2+ SMSCs, the global deletion of postnatal Prrx1+ cells in mice did not lead to craniosynostosis or any other craniofacial phenotype (Wilk et al., 2017). However, ablation of Prrx1+ cells in the embryonic stage of gestation resulted in incomplete calvarial bone formation, indicating that Prrx1+ SMSCs mainly function in the earlier stage of calvarial bone development (Wilk et al., 2017). Ctsk+ mesenchyme has been shown to contribute to long bone fracture healing. Patients with CTSK mutation display abnormal suture and craniofacial bone development including delayed closure of fontanels, hypoplastic premaxilla and obtuse mandibular angle (Debnath et al., 2018; Otaify et al., 2018), yet Ctsk+ SMSCs functions in calvarial bone repair have not been tested. Hhip+ SMSCs were also required for normal coronal suture development. *Hhip* knockout (KO) mice displayed coronal suture dysgenesis, characterized by the reduced or absent overlap of frontal and parietal bones seen in wildtype mice with little or no intervening suture mesenchyme, resulting in more closely apposed OFs in the coronal suture (Holmes et al., 2021). Additionally, Six2+ SMSCs were reduced in the coronal suture of E14.5 and E15.5 embryos from a mouse model of Saethre-Chotzen syndrome (*Twist* ^*+/−*^ *; Tcf12* ^*+/−*^) with coronal synostosis, suggesting the potential functions of Six2+ SMSCs in suture patency (Farmer et al., 2021).

## 3 FGF signaling

**FIGURE 2:**
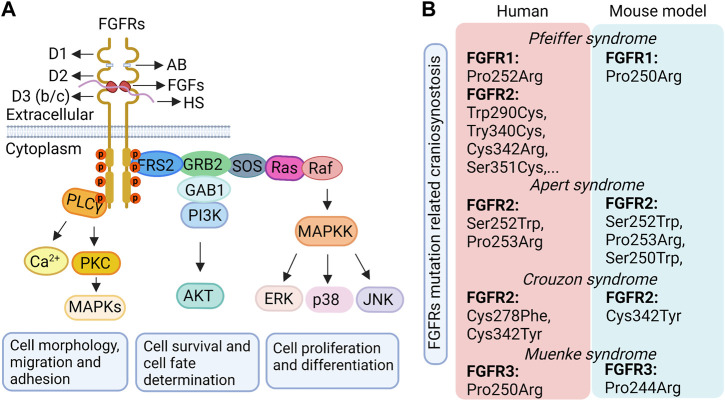
The classical FGF pathway **(A)** and the craniosynostosis-related syndromes caused by FGFR mutations in humans and related mouse models **(B)**. D1, D2, D3, immunoglobulin (Ig)-like domains 1, 2, 3; AB, acid box; HS, heparin/heparan sulfate; FGFs, fibroblast growth factor ligands; FGFRs, fibroblast growth factor receptors.

FGF signaling is a conserved, fundamental pathway that plays distinguished roles in embryonic development and organogenesis, metabolism homeostasis, tissue repair and regeneration, and tumor angiogenesis through the regulation of numerous cellular functions such as cell proliferation, pluripotency, migration, survival and differentiation (Boilly et al., 2000; Cao et al., 2013; Moosa and Wollnik, 2016; Mossahebi-Mohammadi et al., 2020). The FGF family includes multiple FGF ligands and receptors (FGFRs) as mentioned above. FGFRs share a highly conserved structure (Figure 2A) consisting of an extracellular domain that contains 3 immunoglobulin (Ig)-like domains (D1, D2 and D3), an acid box linker region (AB/linker) between D1 and D2, a single transmembrane domain, and a split cytoplasmic tyrosine kinase domain (Wang et al., 1995; Gong, 2014). The FGF binding sites are primarily regulated by the D2 domain, the linker region of D2/D3, and the N-terminus of D3 (Hunter, 2000; Schlessinger, 2000; Gong, 2014; Moosa and Wollnik, 2016; Farrell and Breeze, 2018). Among them, the linker region of D2/D3 is associated with regulating the affinity regulation of both FGFs and heparin/heparan sulfate (HS) (Johnson and Williams, 1992; Mohammadi et al., 2005). Additionally, the specificity of FGF binding is primarily modulated by the alternative mRNA splicing of the C-terminal half of the D3 domain in FGFRs, which generates different FGFR isoforms (McKeehan and Kan, 1994; Wang et al., 1999). For FGFR1-3, the D3 domain includes 3a and 3b or 3c domains and is encoded by exons 7 to 9. The N-terminal half of D3, named 3a, is encoded by exon 7, whereas the C-terminal half containing 3b or 3c is encoded by the alternative use of either exon 8 or 9, which generates the 3b and 3c isoforms of FGFRs, respectively (Johnson and Williams, 1992; Werner et al., 1992; Orr-Urtreger et al., 1993; Cheon et al., 1994). These two different isoforms endow FGFRs with different tissue-expression specificity and ligand-binding affinity. For example, the 3b isoform is predominantly expressed in epithelia tissues, whereas the 3c isoform is mainly expressed in mesenchymal tissues. Ligands activate either the epithelial or mesenchymal FGFR isoforms, with the exception of FGF1, which activates both isoforms (Johnson et al., 1991; Beenken and Mohammadi, 2009; Gong, 2014). Unlike FGFR1-3, FGFR4 has only one isoform (3b) because it contains only one exon encoding the C-terminal half of D3 (Kostrzewa and Müller, 1998). The other alternatively spliced FGFR isoforms are lacking the D1 and/or AB/linker domains (Johnson et al., 1990; Eisemann et al., 1991). The presence or absence of D1 is associated with FGFR autoinhibition rather than their ligand binding activity (Johnson et al., 1990; Chellaiah et al., 1999; Olsen et al., 2004; Kalinina et al., 2012). The FGFR isoforms lacking D1 or AB/linker domains promote the affinity of FGFR for FGFs and enhance the capacity of FGF signaling (Xu et al., 1992; Shi et al., 1993; Wang et al., 1995; Roghani and Moscatelli, 2007).

FGF ligands can induce FGFR dimerization by binding to the extracellular domain of the inactive FGFR monomer. This dimerization subsequently results in the two intracellular kinase domains of the paired FGFRs phosphorylating each other on specific tyrosine residues to activate the FGFR. The activated FGFR then further activates a complex cascade of intracellular signaling events through several downstream pathways, including the Ras-MAP kinase pathway (ERK1/2, p38 and JNK kinase), the PI3 kinase/AKT pathway, and the phospholipase Cγ (PLCγ) kinase pathway (Figure 2A). The activity of these different downstream pathways depends on the cell type with the exception of the Ras-MAP kinase pathway which is activated in almost all cell types (Moosa and Wollnik, 2016). Generally, the Ras-MAP kinase pathway, the main downstream pathway of FGF signaling, is associated with cellular proliferation and differentiation; the PI3 kinase/AKT pathway is associated with cellular survival and cell fate determination and, occasionally, cell polarity; and the PLCγ kinase pathway impacts cell morphology, migration, and adhesion (Teven et al., 2014). Most of these downstream phosphorylation transduction pathways target transcription factors within the nuclei to influence cell proliferation, stemness, migration, survival, and differentiation by regulating gene expression (Moosa and Wollnik, 2016).

FGF signaling contributes to the development of most craniofacial structures, such as the development and outgrowth of the facial primordia, craniofacial skeletogenesis, palatogenesis, as well as development of submandibular salivary gland, teeth, eye lids, craniofacial muscles, and muscular tongue (Nie et al., 2006; Prochazkova et al., 2018; Weng et al., 2018). Perturbation of FGF signaling is involved in various craniofacial abnormalities, including facial or palatal cleft, midface agenesis, mandibular hypoplasia, open eyelids at an early postnatal stage, and craniosynostosis (Ibrahimi et al., 2004; Rice et al., 2004; Wang et al., 2013; Prochazkova et al., 2018; Ray et al., 2020; Xu et al., 2020).

## 4 FGF signaling in cranial sutures

Throughout cranial suture, SMSCs participate in cranial bone growth and development, homeostatic maintenance, injury repair, and cranial suture patency or fusion, which are precisely orchestrated by fine-tuned signals. Studies on patients with syndromic craniosynostosis and a series of mouse studies have indicated a pivotal role of FGF signaling in the development of cranial sutures.

FGF ligands and FGFRs of FGF signaling have distinct spatiotemporal expression patterns in the cranial sutures and SMSCs, depending on their specific functions. FGF ligand family, including FGF2, FGF4, and FGF18, plays important roles in embryonic or postnatal cranial suture development (Moosa and Wollnik, 2016). Among these, FGF18 is the first to be detected in calvarial mesenchymal cells and is later expressed in the osteogenic mesenchyme and differentiated osteoblasts on the endosteal and periosteal surface of skull bones (Moosa and Wollnik, 2016). *Fgf18*-deficient mice exhibited delayed suture closure with decreased proliferation of osteogenic mesenchymal cells and delayed terminal differentiation of osteoblasts (Ohbayashi et al., 2002). The spatiotemporal distribution of FGF2 was distinct among different cranial sutures. For example, *FGF2* expression was significantly higher in posterior frontal SMSCs and the underlying dura than in sagittal SMSCs and the underlying dura during the onset of PFS fusion in mice (Gosain et al., 2004). Of note, Mehrara et al. observed that, FGF2 expression dramatically increased in PFS tissues throughout the process of PFS fusion and reduced after PFS fusion, suggesting that FGF2 benefits PFS fusion (Mehrara et al., 1998). However, *FGF2* expression in the sagittal suture tissues was minimal all times (Mehrara et al., 1998). In rat organ culture studies, PFS treated with FGF2 showed significantly increased fusion on the dura side of the suture compared with the non-treated controls (Moursi et al., 2002). In addition, increasing FGF2 activity also induced coronal suture fusion in rats and mice (Iseki et al., 1999; Greenwald et al., 2001).

In addition to FGF2, FGF3 and FGF4 also play crucial roles during cranial suture development. When the ectopic expression of FGF3 and FGF4 were induced by retroviral insertion in the cranial suture region of mice, extensive premature closure was observed in the cranial sutures, including the metopic, sagittal, coronal, interparietal/occipital and intermaxillary sutures (Carlton et al., 1998). The *ex vivo* culture of E15 mouse calvarial explants with FGF4 bonded beads showed that FGF4 accelerated sagittal sutural closure when beads were inserted in the osteogenic fronts but not when the beads were inserted in the mid-sutural mesenchyme (Kim et al., 1998). Additionally, in humans, an *FGF9* missense mutation led to craniosynostosis with multiple synostosis. This phenotype was mimicked in mice with a spontaneous heterozygous *FGF9* mutation, suggesting that FGF9 plays an important role during cranial suture development (Murakami et al., 2002; Rodriguez-Zabala et al., 2017). Numerous studies have shown that FGFRs, mainly FGFR1-3, are also indispensable in the regulation of cranial suture development. *FGFR1* is primarily expressed in the osteoblast and mesenchyme of the calvarium and is associated with osteoprogenitor differentiation. *FGFR2* is mainly expressed in proliferating osteogenic stem cells and is involved in regulating cell proliferation. Accordingly, in mice, the onset of osteoprogenitor differentiation in the coronal suture is preceded by the downregulation of *FGFR2* expression and the upregulation of *FGFR1* expression (Iseki et al., 1999). Consistently, the expression of a dominant-negative *FGFR1* gene in rat calvaria inhibits suture fusion (Greenwald et al., 2001). These data indicated that the gradients of *FGFR1* and *FGFR2* expression may play important roles in balancing the proliferation and differentiation of osteoprogenitor cells in the cranial suture (Ornitz and Marie, 2002). Iseki et al. also found that *FGFR1* expression was downregulated following the upregulation of osteoblast differentiation markers in mice, indicating that FGFR1 is related to the osteogenic differentiation process but is not involved in maintaining the differentiation stage (Iseki et al., 1999). However, the detailed mechanism needs to be further studied. *FGFR3*, which is expressed at a later stage than *FGFR1* and *FGFR2* in mice, is expressed at low levels in the OFs of suture, and is also expressed in the chondrogenic regions of the skeletogenic membrane, including a thin plate of cartilage underlying the coronal suture (Iseki et al., 1999), suggesting a dual role of FGFR3 in both osteoblasts and chondrocytes during mouse skull development. The *FGFR4* expression has been confined to the cranial musculature (Iseki et al., 1999), while its specific role in cranial suture development remains unknown.

To dissect the mechanisms underlying FGF-FGFR-mediated cranial suture development, the downstream pathways of FGF-FGFR-mediated signaling including Ras-MAP kinase, PI3 kinase-AKT and PLCγ-PKC pathways were studied. Blocking of the ERK pathway using an inhibitor (PD98059) repressed FGF2-induced cranial suture closure in cultured mouse calvaria, and decreased osteoblast differentiation (Kim et al., 2003). Repression of p-ERK1/2 activity in *FGFR2* ^*+/S252W*^ mutant mice using U0126 significantly inhibited craniosynostosis (Shukla et al., 2007). A study from Holmes et al. showed that p-AKT and p-p38 were increased in the calvarial tissues of newborn *FGFR2* ^*+/S252W*^ mutant mice (Holmes et al., 2009). Wang et al. discovered that compared with controls, *FGFR2* ^*+/P253R*^ mutant mice had increased levels of p-p38 and p-ERK1/2 in the neurocranium, together with enhanced osteogenic differentiation and reduced proliferation but without apoptosis changes in the coronal suture (Wang et al., 2010). However, p-AKT and PKCα were not obviously changed in these mutant mice (Wang et al., 2010). Additionally, increased p-ERK1/2 were found in the prematurely fused coronal suture of *FGFR2c* ^*+/C342γ*^ gain-of-function mutant mice, along with enhanced cellularity and dysregulated differentiation of osteoblasts (Pfaff et al., 2016). Together, these results suggest that the downstream pathways of FGF-FGFR-mediated signaling, especially the Ras-MAP kinase pathway, play important roles in FGF-FGFR-mediated cranial suture development and are context-dependent. However, further studies are needed.

## 5 FGF signaling in craniosynostosis

Given the complicated functions of FGF signaling in cranial sutures, it is no surprise that its dysfunction gives rise to various craniofacial related diseases. Familial studies have revealed that patients with craniosynostosis primarily show a gain-of-function mutation within the gene region of FGFRs responsible for the linker between the D2 and D3 extracellular domains (Figure 2B). This type of mutation may activate FGF signaling either in a ligand-dependent manner by changing the affinity and specificity of FGFRs to their corresponding FGF ligands (Ibrahimi et al., 2001; Ibrahimi et al., 2004; Moosa and Wollnik, 2016), or in a ligand-independent manner by enhancing FGFR dimerization (Kan et al., 2002; Moosa and Wollnik, 2016). As a result, the proliferation, differentiation and/or apoptosis of cells in the cranial suture are changed resulting in craniosynostosis (Passos-Bueno et al., 1999; Teven et al., 2014). For instance, in humans, Apert syndrome, characterized by premature fusion of the bilateral coronal sutures and severe syndactyly of the feet and hands, is caused by Ser252Trp and Pro253Arg mutations of the *FGFR2* gene in the D2-D3 linker region, which leads to *FGFR2* gain-of-function in a ligand-dependent manner (Slaney et al., 1996; Ferreira et al., 1999; Ibrahimi et al., 2001; Andreou et al., 2006; Ko, 2016; Kunwar et al., 2017). Pfeiffer syndrome in humans, which shows similar craniofacial anomalies to those seen in Apert syndrome along with big toes and broad radially deviated thumbs (Giancotti et al., 2017), is due to a mutation in either *FGFR1* (Pro252Arg) or *FGFR2* (Trp290Cys, Try340Cys, Cys342Arg, or Ser351Cys, *etc*.) (Azoury et al., 2017). The *FGFR1* (Pro252Arg) mutation leads to a bulkier residue that enhances the binding affinity of the receptor to the ligand to increase receptor activation (Ibrahimi et al., 2004). The *FGFR2* mutation mainly causes the ligand-independent activation of the receptor by leading to an unpaired cysteine residue that forms an intermolecular disulfide bond (Cornejo-Roldan et al., 1999; Lajeunie et al., 2006). Muenke syndrome in humans, characterized by craniosynostosis with uni- or bicoronal synostosis, comes from an *FGFR3* Pro250Arg mutation resulting in the increased binding affinity of FGFR3 to its ligand (Muenke et al., 1997; Ibrahimi et al., 2004), such as FGF9, by the substitution of a bulkier residue. In addition, other craniosynostosis syndromes, including Jackson-Weiss syndrome and Crouzon syndrome, are also caused by gain-of-function mutations in the D2-D3 linker region of FGFR1 or FGFR2 in a ligand-dependent or independent manner (Moosa and Wollnik, 2016). However, *FGFR2* mutations contribute to the majority of craniosynostosis syndromes in humans (Ornitz and Marie, 2002).

Results from animal studies have further supported the critical roles of FGF signaling in cranial suture development. As mentioned above, in mice, *FGF2*, *FGF3*, and *FGF4* overexpression lead to suture synostosis, and *FGF18* loss-of-function results in delayed suture closure. Gain-of-function mutations of *FGFR1* and *FGFR2* in mice also impact cranial suture development (Figure 2B). *FGFR1* P250R mutation in mice, which is orthologous to the Pfeiffer syndrome mutation (*FGFR1* P252R) in humans, leads to the premature fusion of calvarial sutures including frontal, sagittal, and coronal sutures (Zhou et al., 2000). *FGFR2* ^*+/S250W*^ transgenic mouse, an Apert syndrome mouse model, showed premature closure of the coronal suture (Chen et al., 2003). Additionally, Wang et al. observed that *FGFR2* ^*+/S252W*^ mutant mice, another Apert syndrome mouse model with *FGFR2* gain-of-function mutation, showed proximate OFs of two parietal bones and abnormal osteoid deposited between them when compared with controls; while the interfrontal suture of mutant mice exhibited a broad gap between the OFs of frontal bones when compared with control ones (Wang et al., 2005). Concomitantly, they found that *FGFR2* ^*+/P253R*^ mutant mice, another *FGFR2* gain-of-function mutation that commonly occurs in patients with Apert syndrome, had cranial features that resembled those shown in *FGFR2* ^*+/S252W*^ mutant mice (Wang et al., 2010). Additionally, *FGFR3* ^*Y367C/+*^ (*FGFR3* gain-of-function) mutant mice also showed partial premature fusion of coronal sutures and impaired frontal bones, suggesting important roles of FGFR3 in suture patency and membranous ossification (Di Rocco et al., 2014). Nevertheless, *FGFR3* ^*P244R/+*^ mutant mice, a model of Muenke syndrome with *FGFR3* gain-of-function, displayed mild skull deformities and rarely showed premature fusion of the coronal suture (Twigg et al., 2009). *FGFR3* KO mice did not show obvious calvarial bone defects (Valverde-Franco et al., 2004). Furthermore, mice with *FGFR3* P244R mutation (equivalent to the human P250R mutation), a genetic model for Muenke syndrome, show a rounded skull and shortened snout with dental malocclusion which are similar to Muenke syndrome features in humans. However, coronal craniosynostosis in human patients is not reliably reproduced in this mouse model (Twigg et al., 2009). This suggests different functions of FGFR3 between mice and humans. Whereas the detailed pathological mechanism underlying FGF/FGFR related craniosynostosis is still poorly understood.

## 6 FGF signaling crosstalks with other signals to regulate cranial suture development

As a pivotal regulatory signaling that functions during cranial suture development, FGF signaling broadly crosstalks with many other transcription factors and signals to orchestrate complicated processes. For example, Twist, a basic helix-loop-helix transcription factor, is expressed in SMSCs and regulates osteoblast differentiation and cells apoptosis (Howard et al., 1997; Yousfi et al., 2002). The haploinsufficiency of *Twist* leads to premature fusion of the cranial suture (Yousfi et al., 2002). In contrast, trisomy at the human *TWIST* locus results in delayed suture closure (Stankiewicz et al., 2001). In addition, the distribution pattern of FGFR2 was changed in the sagittal suture of *Twist* ^*+/−*^ mice when compared with wildtype mice (Rice et al., 2000). In wildtype mice, *FGFR2* was mainly expressed in osteoblasts of the OFs and weakly and diffusely expressed in SMSCs in the sagittal suture. However, in *Twist* ^*+/−*^ mice, FGFR2 localized more in the mid-sutural mesenchyme. Additionally, their study also displayed that exogenous FGF2 in the mid-suture mesenchyme stimulated Twist expression in *ex vivo* cultured sagittal sutures to inhibit osteoblast differentiation of suture mesenchyme (Rice et al., 2000). Accordingly, they brought the point that Twist could be a potential transcriptional regulator that modulates the inhibitory effects of FGF2 on osteoblast differentiation (Rice et al., 2000). MSX1 and MSX2, which are homeobox-containing transcription factors, are expressed in the mesenchyme and are involved in the differentiation of NC-derived calvarial bones (Ornitz and Marie, 2002). *MSX2* overexpression in mice or mutation in humans leads to craniosynostosis with an increased osteoprogenitor population. Conversely, *MSX2* haploinsufficiency in mice or humans results in reduced cell proliferation and delays suture closure, together with defective skull bone ossification (Ornitz and Marie, 2002). In mouse and rat calvarial cells, MSX2 was identified as an upstream factor to inhibit the osteogenic activity of FGF2. In addition, FGF4 could enhance *MSX1* expression and cell proliferation. Runx2/Cbfa1 is a key transcription factor to initiate mesenchymal stem cells to differentiate into osteoblasts. Heterozygous loss-of-function mutation of *RUNX2* in humans is associated with cleidocranial dysplasia (CCD) with open fontanelles. Similarly, open fontanelles were also observed in *Runx2* ^+/−^ mutant mice with disturbed sagittal suture formation (Qin et al., 2019). Interestingly, Qin et al. found that *Runx2* loss-of-function in mice led to reduced proliferation and condensation of SMSCs (Qin et al., 2019). They further discovered that the expression of FGF signaling related genes, including *FGFR1*, *FGFR2* and *FGFR3*, was significantly reduced in the suture regions but not in the calvarial bone tissues of *Runx2* ^+/−^ mutant mice. In addition, the expression of several other signaling factors was also decreased, such as Gli1, Ptch1 and Ihh in Hedgehog signaling, and Tcf7, Wnt10b and Wnt1 in Wnt signaling, suggesting the important role of coordinated signaling in SMSCs during cranial suture development (Qin et al., 2019). Additionally, TGF-β1, similar to FGF2, is upregulated in the PFS mesenchyme and dura during the closure of the PFS (Most et al., 1998; Gosain et al., 2004). Sasaki et al. found that FGF acts downstream of TGF-β signaling to promote cranial NC cell proliferation during frontal bone development, and FGF2 could rescue the proliferation defect caused by *Tgfbr2* mutation (Sasaki et al., 2006). BMP signaling is required for osteoblast differentiation and may function in concert with FGFs to control calvarial bone development (Schliermann and Nickel, 2018). Moreover, Jiang et al. revealed that BMP2 was crucial for the FGF2-dependent later-stage osteoblastic differentiation of cranial suture cells that were isolated from bone fragments around the coronal and sagittal sutures of newborn rats. They found that the expression of BMP2 could be initiated by FGF2 in a time and dose-dependent manner (Jiang et al., 2015). FGF2 treatment may reduce the early osteoblast differentiation marker, *Col1a1*, expression, while enhancing the late markers (*Alp*, *Ocn* and *Bsp*) expression to promote mineralization. BMP2 inhibition could reduce the induction of FGF2 to later-stage osteoblast differentiation of cranial suture cells (Jiang et al., 2015). Recently, Min Swe et al. found that Lrp5 and Lrp6, co-receptors of Wnt/β-catenin signaling, were aberrantly activated in the developing coronal sutures of Apert syndrome (*FGFR2* ^*+/S252W*^) mouse models (Min Swe et al., 2021). *Lrp5* and *Lrp6* knockdown dramatically decreased osteoblast differentiation markers (*Runx2*, *Col1a1*, *Ocn* and *Alp*) expression in cultured cells isolated from coronal sutures of *FGFR2* ^*+/S252W*^ mice, indicating an interaction between FGFR2 and Wnt/β-catenin signaling (Min Swe et al., 2021). The FGF signaling pathway has also been found to interact with other signaling pathways, such as Notch, Hedgehog, Hippo, and mechanical signaling pathways, which also play important roles in the proliferation, differentiation, and apoptosis of osteoprogenitors and osteoblasts (Byun et al., 2014; Li et al., 2020; Zhao et al., 2021; Zhao et al., 2022). However, whether these interactions play a role during cranial suture development and how they function in SMSC proliferation and differentiation remain largely unknown.

## 7 Conclusion and future prospectives

SMSCs are located in the cranial suture and are characterized as a heterogeneous stem cell population. SMSCs have a distinct ability to self-renew and differentiate into multiple cell lineages, including osteoblasts and chondrocytes, in a tempo-spatial dependent manner. SMSCs make significant contributions to craniofacial development, suture patency maintenance, and cranial bone repair and regeneration. It has been established that the proliferation, differentiation, and apoptosis of SMSCs and their derivatives are associated with multiple factors and signaling pathways, including Twist, Msx1/2, Gli1, Axin2, as well as FGF, Wnt, Hedgehog, NOTCH, Hippo, and mechanical signaling to orchestrate SMSCs and cranial suture development. Any perturbation of these factors and pathways may open a window for an array of diseases caused by abnormal development of SMSCs and cranial sutures, especially those characterized by craniosynostosis.

The FGF signaling pathway is a highly conserved, fundamental pathway that regulates numerous processes, ranging from embryonic development and organogenesis to adult tissue repair and regeneration. Dysfunction of FGF signaling has been linked to multiple human diseases (Xie et al., 2020), such as dwarfism syndrome, chronic kidney diseases (CKD), various tumors, and craniosynostosis, Clinical and experimental evidence showed that FGF signaling controls cranial suture development likely through modulating a balance among the proliferation, differentiation, and apoptosis of cranial sutural cells in a tissue- and stage-specific manner, but this still needs further study. Notably, most of the FGF signaling related craniosynostosis diseases are thought to be *FGFR* gain-of-function mutations, either in a ligand-dependent manner by altering the ligand-binding affinity or specificity, or in a ligand-independent manner through stabilizing intermolecular disulfide bonds to constitutively activate the receptor and signaling. Reports have shown that the majority of craniosynostosis syndromes were related to *FGFR2* gain-of-function mutations. However, it is worth noting that different sutures respond to FGF-FGFR signaling differently. Compared with other sutures, craniosynostosis mainly occurs in the coronal sutures in *FGFR* gain-of-function mutant animal models, such as *FGFR2* ^*+/S250W*^ and *FGFR2* ^*+/S252W*^ transgenic mice, and *FGFR3* ^*Y367C/+*^ mutant murine models (Wang et al., 2005). This may be due to the spatiotemporal- and tissue-specific expression pattern of FGFs and FGFRs as well as the different embryogenic origins of suture cells that have different responses to FGF signaling. As mentioned above, SMSCs of the coronal suture are mostly derived from mesodermal cells while the frontal and sagittal sutures are mainly derived from NC cells. NC-derived mesenchyme showed higher intrinsic proliferation and osteogenic abilities than mesoderm-derived mesenchyme, and expression of FGF18 and FGFR3 was higher in NC-derived MSCs than in mesoderm-derived MSCs. This leads to varying responses by cells of different embryonic origins to FGF signaling that is associated with cell proliferation, differentiation and apoptosis, representing an interesting field for further studies. Robust studies have been performed to explore the pathological processes of craniosynostosis and significant progress has been made. However, the detailed molecular mechanism of how FGFR mutations impact downstream molecules and signaling pathways leading to various diseases and how such molecules and pathways provide feedback to regulate FGF signaling is still poorly understood due to the intricate nature of FGFs and FGFRs and their multiple downstream pathways, as well as complicated SMSCs. Taken together, these findings described the populations and characteristics of SMSCs and indicated the complicated and critical roles of the FGF pathway in the development of the cranial suture and SMSCs, meanwhile highlighting the significance of studying the FGF pathway in cranial suture development and related diseases, especially craniosynostosis. We also summarized the broad crosstalk between the FGF pathway and other factors and pathways during cranial suture development and related diseases, which sheds light on the mechanistic studies of FGF-related craniofacial diseases. However, further investigations of the interactions and functions of the SMSC population, and the detailed mechanism underlying how environment transcription factors and signaling pathways coordinate with FGF signaling to orchestrate cranial suture and SMSCs development or cause suture-related diseases are urgently demanded. These may contribute to the development of therapeutic interventions with SMSCs for cranial diseases. In summary, the FGF signaling pathway has pivotal functions in cranial suture and SMSCs development and warrants further investigation in the mechanisms underlying cranial suture development and related diseases with the hopes of improving current diagnostic and therapeutic options.

## Abbreviations

SMSC, Suture mesenchymal stem cell; NC, neural crest; OFs, osteogenic fronts; CCD, Cleidocranial dysplasia; FGFRs, fibroblast growth factor receptors; MSCs, mesenchymal stem cells; PFS, posterior frontal suture; MAPK, mitogen-activated protein kinases.
